# Kenneth Gergen’s concept of multi-being: an application to the nurse–patient relationship

**DOI:** 10.1007/s11019-019-09897-4

**Published:** 2019-04-15

**Authors:** Mareike Hechinger, Hanna Mayer, André Fringer

**Affiliations:** 1 Department of Nursing Science, University of Vienna, Alser Straße 23/12, 1080 Vienna, Austria; 2 School of Health Professions, Institute of Nursing, Zurich University of Applied Sciences ZHAW, Technikumstrasse 81, 8400 Winterthur, Switzerland

**Keywords:** Nurse–patient relationship, Nurses, Patients, Interaction, Homecare, Dyad

## Abstract

The nurse–patient relationship is of great significance for both nurses and patients. The purpose of this article is to gain an understanding of how the individual is constituted through a focus on the execution of the patient’s and nurse’s role in the joint relationship. The article represents a social-constructionist consideration using Kenneth Gergen’s concept of multi-being. Gergen’s notions of the self as a multi-being focuses on the individual’s relational character through former relationships and social interactions. Gergen’s concept is applied onto nurses and patients as individuals to gain an understanding of the broader institutional and social context of each role and their interactions within the nurse–patient relationship. The article focuses on the nurse–patient relationship in general with regard to specific challenges in the home care setting. Various demands and experiences from a myriad of past relationships merge as potential actions for nurses and patients during the forming of a relationship. Nurses as multi-beings see themselves confronted with guidelines and legal conditions, their own as well as the patients’ expectations and the actual possible forming of a relationship in the light of daily nursing care. Patients as multi-beings experience an extended social environment that comprises the nurse–patient relationship while simultaneously having to cope with illness and increasing care dependency within their own homes. Discrepancies can be observed in the relationship with regard to the inherent human qualities, the demands of forming a relationship, *and* the actual relationship arising due to framework conditions.

## Introduction

The nurse–patient relationship is characterized by nurses working with vulnerable individuals who are dependent on care in situations that are often intimate in nature. The two individuals meet in a specific context, each having their own expectations, needs, and tasks, apart from different past experiences. They each come from individual backgrounds that involve origin, ethnicity, culture, religion, generation, and a socio-economic situation (Ujhely 1968). They, together, form a relationship where one plays the role of caregiver and the other, care receiver.

In the homecare setting the nurse–patient relationship takes place in the patient’s private space and this entails a certain amount of trust, dependency, vulnerability, and intimacy (Holmberg et al. 2012; Angus et al. 2005; England and Dyck 2011; Büscher 2007). When it comes to receiving professional care in their own homes, patients experience difficulties in maintaining their dignity, integrity, and autonomy (Holmberg et al. 2012). Similarly, it is challenging for nurses to switch roles between being a guest and a professional during home care (Oresland et al. 2008). The nurse–patient relationship in the German-speaking homecare setting differs from that of most English-speaking countries in that there are no mandatory professional guidelines that clarify the form of these relationships. The nurses can independently form a relationship within the legal context. This forming of the nurse–patient relationship, is aside from the professional approach, a mainly individual one. The nurses bring their own personal attitudes and characteristics into their work (Büscher 2007); where some nurses tend to engage merely professionally, others prefer to involve themselves by bringing more personal characteristics into the work-related relationships. Sometimes, nurses tend to engage beyond the professional boundaries of their work, irrespective of their educational background (Hechinger 2016). To involve oneself in such a manner within a nurse–patient relationship is discussed occasionally within the German professional context, but is only regulated by the nurses’ employers. In literature pertaining to English-speaking countries, this could be described as over-involved behaviour (Nursing and Midwifery Board of Australia 2010; Nursing Council of New Zealand 2012; Canadian Nurses Association 2017; Nursing & Midwifery Council 2015; National Council of State Boards of Nursing 2014).

Thus, the individuals executing the roles of nurse and patient have different motives and expectations that reflect on their actions within the relationship. A social-constructionist perspective of individuals building their understanding of the world together, could promote an insight into the complex interplay of the nurse–patient relationship. Since such an approach is currently non-existent it is necessary to understand the elements of the nurse–patient relationship by exploring each of the roles. Therefore, this social-constructionist perspective is transferred onto nurses and patients by applying Gergen’s (2011) concept of the individual as a relational being. The terms “nurses” and “patients” comprise individual persons executing the respective socially constructed role. In the case of “nurses”, the term refers to healthcare professionals, regardless of their educational background, as persons with varied educational backgrounds ranging from none to an academic degree provide care for patients. International literature has been used to outline the following considerations which are supplemented by the first author’s experiences of the German homecare system that serves as a springboard for developing the theoretical discourse.

Gergen (2011) describes the individual as a relational being, a so-called multi-being, and thus, being constituted through his or her former relationships with other individuals. These former relationships leave traces (such as habits and experiences) that function as countless potential actions, so-called potentials, which are brought forth in actual social interactions. He states that “it is not individual ‘I’s who create relationships, but relationships that create the sense of ‘I,’ […]. Rather, ‘I’ am just an I by virtue of playing a particular part in a relationship” (Gergen 1991, p. 157). Nurses and patients as individual multi-beings are each constituted through their former relationships, and they encounter each other as protagonists in their jointly formed relationship.

The central aim is to explore these former relationships given the multiple potentials that can merge in the nurse as an individual in a professional capacity and the patient as an individual who needs assistance due to health deficiencies. Initially, the focus will be on understanding the individuals’ constitution. Later, based on this, the nurse–patient relationship will be explored. As it is not possible to detail each and every past relationship or every possible development of a potential, this theoretical discourse focuses only on the individuals’ common aspects pertaining to their roles as nurses and patients. The aspects relating to the individual’s multi-being as a human in general are described elsewhere (see also Gergen 2011).

This article is divided into three parts. First, Gergen’s concept of the individual as a multi-being is explained. Second, the notions regarding the multi-being are transferred separately onto the nurse and patient, and they focus on the challenges of homecare services. Third, the studied notions are combined and transferred onto the actual interactions in a nurse–patient relationship.

## The individual as multi-being

Various authors have formulated ideas that can be summarized under the term “social constructionism”. Although there is no concrete understanding of the term the resemblance is that knowledge is obtained from social processes and actions (Burr 2015). Consequently, various “realities” or rather perspectives of reality—can exist at the same time (Berger and Luckmann 1991; Gergen 2015; Schuetz 1945). Similarly, in the nurse–patient relationship, each nurse and patient is embedded in their individual social context and they construct their understanding from past social relations and interactions.

Following the paradigm of social construction, Berger and Luckmann (1991) as well as Gergen (2011) focus on the perspective of the individual being constituted through social interactions but with a divergent understanding of reality. With their sociological background Berger and Luckmann (1991) carried forward Schuetz’s (1945) thinking on multiple realities resulting from social interactions, even though Schuetz also described “objectifications” of reality which referred to people sharing their understandings. According to Berger and Luckmann (1991) there is a dualism of objective and subjective reality with the objective reality being internalized by the individual. Upon birth, people enter a world already constructed by their predecessors that then becomes an objective reality for them. In contrast, Gergen (2011, 2015) proposes a consistent relativist position in which the individual constantly negotiates meaning with others through co-action and thus, an intersubjective reality. Co-action is understood as a process of collaborative and coordinated action resulting from a relationship. Gergen’s considerations on co-action relate to Shotter’s (1980) works and Blumer’s (1986) symbolic interactionism. As a consequence, Gergen (2015) describes the basic constructionist idea as “*nothing* is real unless people agree that it is” (p. 5). In his opinion there are always material objects and events occurring (such as a person’s death) around us but the name or meaning is constructed in a process of co-action. When people try to describe an event such as a death, they do so by referring to a certain tradition of knowledge. Biologically speaking, the person may have died, for example from an illness or an accident. But if speaking from a religious point of view, the deceased has gone to heaven. The constructionist view enables one to appreciate the different kinds of knowledge (Gergen 2015). Although Gergen’s thoughts may derive from a psychological context they hold interesting aspects for the social and nursing sciences because his way of thinking promotes an understanding and reflection of a nurse’s and the patient’s behaviour. His considerations of the individual as a relational being are fundamental to this article in terms of the further exploration of nurses and patients as multi-beings within their joint relationship.

Gergen (2011) promotes the view of the self as a multi-being with a relational character. Residues or resources arise from each relationship in the form of potential actions, such as language, facial expressions of emotions, gestures, or behavioural patterns. The emerging residues become potentials that are unequally emphasized since some are more pronounced than others. While some can only be a hint of a possibility, others are well-practiced residues, such as habits or skills. When two people interact, only potentials of their respective selves become obvious while the personality as a whole remains hidden. It is only with the occurrence of multiple interactions within the encounter that the other’s additional potentials can be discovered and absorbed. Gergen describes three sources for absorbing potentials in relationships: (1) Using the actions of another person as a *model* for one’s own actions; (2) *becoming somebody* through the experience of different roles in different relationships (such as child, father, employee); and (3) participating in *interactive scenarios* within a relationship (such as learning to dance). The examples for these three sources in the nursing context could be: (1) a student imitating the mentor to learn specific techniques; (2) experiencing the roles as a professional, a colleague, and/or an employee; and (3) learning to execute a nursing intervention with the patient. As Gergen (2011) puts it: “In sum, all meaning/full relations leave us with another’s way of being, a self that we become through the relationship, and a choreography of co-action. From these three sources, we emerge with enormous possibilities for being” (p. 137). Consequently, with these potentials that are incorporated in the individual’s self, the self is well equipped for further social interactions.

Gergen illustrates a world of co-constitution where an individual emerges from a relationship but through interactions continuous to stay in relationships with other individuals. Boszormenyi-Nagy and Spark (2014) describe this phenomenon using their concept of invisible bonds existing among individuals that *influence* our being. Gergen (2011), however, criticizes the common understanding of cause and effect that is promoted by speaking of influences and effects. He believes that cause and effect are intertwined and defined reciprocally. Furthermore, he takes on a relativist position that considers potentials from past relationships in terms of relational confluence. The concept of confluence has similarities to Bourdieu’s ideas of habitus as a set of dispositions that emerge from determining structures, such as family and education (Bourdieu 1977). As Gergen (2011) understands that one’s actions are a result of past relationships, he criticizes the idea of the individual having free will, as inherent in western culture. In his opinion there is no “free” decision that exists in an interaction because individuals are defined by their experiences of having been part of several past relationships and therefore his/her “decision” is as well. He sees the individual as a bounded being and thus, being separate and singular. His considerations about the bounded being were used as a starting point to transition onto thoughts on the relational being. In his opinion, it is neither true nor false to see the world in terms of determinism and bounded beings, it is just a social construction; “a tradition that has become so commonplace that we forget that it is a human creation” (Gergen 2011, p. 27).

In the context of the nurse–patient relationship both the nurse and the patient have to be seen as multi-beings with past social interactions having shaped their respective selves. From a social constructionist viewpoint the nurse–patient relationship is a fluid, dynamic process that exists because nurses and patients have experienced hundreds of other relationships (Swauger 2016) before they even meet for the first time.

## The nurse as multi-being

**Fig. 1:**
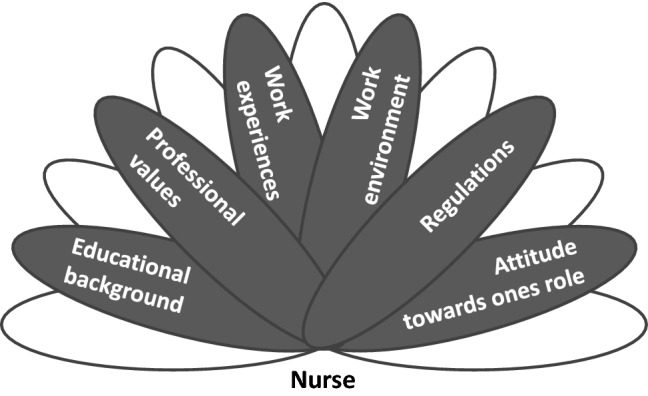
Schematic diagram of nurses as multi-being. Own diagram: 2019

Having transferred the concept of Gergen’s (2011) relational being onto the nurse’s role relations within the following aspects mainly shape the multi-being: educational background, professional values, work experiences, work environment, regulations to form the relationship, and the attitude towards the role. Figure 1 shows a schematic diagram of the identified aspects that play a role in a nurse’s multi-being. The aspects comprise past experiences and relationships from which potentials have been gained. Additionally, the figure shows empty potentials since every individual has countless other potentials shaping his or her multi-being that have been derived from aspects such as origin, culture, religion, or socio-economic background.

### Educational background

Individuals gain role-specific knowledge through social interactions (Gergen 2015), which in the case of nurses is educational training and ongoing nursing practice. Nevertheless, the content available as part of the educational training may not necessarily correspond to the information internalized by the student. Gergen (2011) illustrates how a student and a teacher mutually create meaning, reason, and value. In a process of co-action, the teacher’s words and actions gain the meaning. The nursing student—as a multi-being—brings into the classroom his/her pre-existing circle of relationships and thus, different skills, deficiencies, values, fears, and enthusiasms. So, the teacher imparts the same information to all the students but the meaning that is given to the words or the probable potentials absorbed from this encounter will depend on the student nurse’s multi-being.

The educational training conveys knowledge that is valid for the date of the training and also comprises the current idea of man. During the last century the training content changed from merely concentrating on the patient’s deficiencies to one that included the patient’s resources, as seen in present day nursing practice. It was not until the twentieth century that the nursing practice developed from being just task-oriented to patient-oriented care, and the therapeutic significance of the nurse–patient relationship was acknowledged (Aranda and Street 1999; Peplau 1991). The specializations within the educational training, such as geriatric care, convey further content and competencies. Working with a particular orientation is approved of as a natural condition. Crowe (2000) describes the process of becoming a nurse as “adhering to the established practices of the nursing culture” (p. 963). In Gergen’s (2011) terms, a student tends to use another nurse as a model to absorb potentials for one’s own actions. The educational training is seen as a starting point because a multitude of experiences in nursing practice are collected thereafter, and the skills continue to improve with an increase in experience (Ujhely 1968; Benner 2001).

### Professional values

Professional values are inherent in nursing practice (Ujhely 1968). These values are created in social interactions (Gergen 2011) which could include interactions with patients and colleagues during the process of becoming a nurse. It is a prerequisite for nurses to be reflective and to engage themselves in caring encounters with patients, and should involve aspects showing an understanding of patient needs; striving for equality within the relationship; and acceptance of the patients’ individuality (Snellman and Gedda 2012). The important attributes of the nurse–patient relationship include understanding, non-judgment, and a positive and fresh attitude (Cleary et al. 1999). For developing trust in the relationship, it is necessary to be honest, trustworthy, engaged, authentic, sensitive, confidential, respectful, and aware of patients’ needs (Dinç and Gastmans 2013). Aranda and Street (1999) describe core behaviours of nurses as “being authentic” and “being a chameleon”. Homecare nurses experience difficulties in having to make a choice between the roles of being professionals and being guests in the patients’ homes (Oresland et al. 2008). This illustrates the necessity of incorporating different or even divergent behaviours during interactions to respond to the specific needs of patients. Gergen (2011) illustrates that “learned” values or behaviours are absorbed as potentials, but the realization of these values differ from one nurse to another. That means that our knowledge and words used in interactions are always coloured by our own values.

In this context, the professional identity has to be considered as it is related to how the nurses’ values and beliefs guide their thinking and actions (Fagermoen 1997). The nurses use various sources for developing their professional identity including the public image, work environment, work values, education, and traditional sociocultural values (ten Hoeve et al. 2014). This indicates that the professional identity develops through a process of social interaction and self-reflection (Fagermoen 1997; Berger and Luckmann 1991). As Gergen (2011) puts it, self-identity is always in motion as it is an ongoing, never-ending process. Thus, nurses do experience a dissonance between their own and others’ expectations *and* their actual experiences of nursing practice during the process of professional socialization (MacIntosh 2003). Referring to Gergen (2011), experiencing of discords is normal due to varying interactions and individual experiences. It is part of everyone’s multi-being to have divergent potentials. It is up to us to value “the myriad potentials for effective co-action across a broad and disparate field of relationships” (p. 137).

### Work experiences

When students transition onto working as nurses, they have in Gergen’s (2011) terms “*become somebody*” since they experience a new role. By gaining a higher level of proficiency nurses experience an increasing independence from abstract principles due to lived experiences and an altered perception of situations that enables a comprehensive understanding of the whole (Benner 2001). An increase in experiences of the nurse–patient interaction encourages nurses to develop their communication skills and adopt a more authoritative attitude (Ujhely 1968). As nurses are expected to assess the patients’ needs, risks, or resources, the patients tend to become objectified from the nurses’ perspective, indicating an asymmetrical aspect in the relationship.

As the patient interactions increase, the nursing students experience both personal and professional growth, in addition to a rise in confidence and self-esteem (Suikkala and Leino-Kilpi 2005). Regarding nurses who have only a few skills for establishing a therapeutic relationship, it is stated that these nurses rely on other ways of forming relationships as learned in their private lives (Pohlmann 2005). This shows how potentials absorbed in the working environment contribute to the individual’s multi-being in general, and vice versa.

During the course of providing patients with support and care, the nurses’ vulnerability tends to rise because of exposure to negative experiences involving patients turning against them. The past negative experiences, as often unforeseen verbal or physical attacks, can cause constant anxiety for nurses during work (Angel and Vatne 2016). Thinking in terms of co-action, Gergen (2011) illustrates that an action, in itself, has no meaning. Another person, in the given context, is required to react with words or gestures so as to create meaning and thus, provide the negative valuation of a verbal or physical attack. The potentials of having experienced such an event can become part of one’s multi-being and can lead to a fear of such events occurring in future.

### Work environment

The place of care provision is important in the nurse–patient relationship (Wiechula et al. 2016). Economic changes have led to the development of the current professional nursing care practice. Nurses experience a great amount of stress and workload with less time for their patients, especially in the homecare setting (Billeter-Koponen and Fredén 2005; Kreutzer and Slotala 2012; Büscher 2007; England and Dyck 2011). Organizational and educational modifications in the nursing profession cause a strain on nurses because they then promote changes in the nurses’ actions and raise moral and ethical questions (Billeter-Koponen and Fredén 2005). Nurses are expected to be effective in their care delivery as care is considered a commodity (Crowe 2000). Economic factors expect the focus to be on the patients’ bodily needs which should result in a “caring for” the patients’ bodies. However, in practice nurses often “care about” their patients (England and Dyck 2011). Caring as a social act is seen to be endangered because it is expected to recede into the background (Watson 2003; Käppeli 2005). This adjustment is associated with the nurse–patient relationship based on a business-oriented contract that is signed at the time of hospitalization or availing of homecare services (Käppeli 2005).

The homecare nurses have several competencies which include the ability to work alone, take decisions independently, improvise considerably due to the environment, and display flexibility because of having several different workplaces (Büscher 2007; Ujhely 1968). They constantly experience working together with the patients’ family members and informal caregivers. Relatives can be perceived as a facilitating or hindering factor (Büscher 2007; Hechinger 2016). Büscher (2007) states that nurses perceive themselves as being in charge and they feel responsible, especially in regard to patients living alone. Their concept of homecare is to work closely with the persons involved in the home environment. However, sometimes in the course of their work nurses feel caught between stools when they are forced toplay mediator between relatives, physicians, and their employer (Büscher 2007). Speaking in terms of Gergen (2011) nurses as multi-beings always carry their potentials of past relationships with them and can bring them into their current work. Nurses have precise ideas of how they want to work but they are limited in what can be achieved due to the prevalent economic factors and regulations.

### Regulations

The regulations governing the nursing profession differ from country to country, consequently each country has its own set of considerations pertaining to the nurse–patient relationship, such as laws, professional guidelines, codes of conduct, or regulations of the employers. Gergen (2011) points out that humans construct standards for judging “good” and “bad”. When interacting with each other, individuals follow patterns of coordination that have derived from these conventions to describe what is acceptable and what is not. During their educational training and working practice, new nurses learn to comply with these requirements as expected by using other nurses as models.

A world-wide known standard for nurses is the code of ethics from the International Council of Nurses (2012). The code emphasizes respect for human rights and is a guideline for ethical conduct. In countries with no specific guidelines or codes of conduct to structure the nurse–patient relationships, the regulations can be established by the nurses’ employers in addition to the nurses defining their own boundaries. The process of defining boundaries is recognized as one that initializes at the start of the nurse’s career and continues throughout his/her entire working life (Hechinger 2016). This process illustrates how the actions of nurses are moulded to become increasingly precise through potentials absorbed from various interactions in their working life. In the first encounter, a nurse may have less-defined boundaries which can lead to negative experiences. Subsequently, they may learn to clarify their own boundaries in future encounters.

In most English-speaking countries, additional regulations have been established using a continuum that comprises a zone of helpfulness within the therapeutic relationship to differentiate the undesired over- or under-involved behaviours. This continuum promotes an ideal of correct behaviour for professional practice (National Council of State Boards of Nursing 2014; Nursing and Midwifery Board of Australia 2010; Nursing & Midwifery Council 2015; Nursing Council of New Zealand 2012). Violations of boundaries such as excessive self-disclosure and acceptance of gifts are seen as transgressions within the relationship (Manfrin-Ledet et al. 2015). The nurse is expected to be responsible and maintain professional boundaries as these “are the spaces between the nurse’s power and the patient’s vulnerability” (National Council of State Boards of Nursing 2014, p. 4). Originating from the perspective of the therapeutic relationship imbalances of power between nurse and patient as following the patient’s vulnerability have been stated (Delmar 2012; National Council of State Boards of Nursing 2014; Shatell 2004). Nevertheless, nurses are encouraged to show involvement while simultaneously maintaining a professional distance (Duppel 2005; Nursing Council of New Zealand 2012; Williams 2001).

Various expectations are linked with the nurse’s role. Often, nurses find themselves caught between their own and the patient’s expectations, guidelines and laws as well as the actual possible forming of a relationship due to economic framework conditions and limited time resources (Attree 2001; Hechinger 2016; Kreutzer and Slotala 2012; MacIntosh 2003). The discrepancies that arise due to the expected professional behaviour, professional identity, and the daily nursing care become obvious. These notions illustrate the contrast in the individual professionalization, thus referring to the individual’s development of professional identity and the professionalization of nursing as a profession. These contradictory demands often provoke role conflicts (Hem and Heggen 2003; Pohlmann 2005) as illustrated in greater detail in the next section.

### Attitude towards one’s role as nurse

The understanding of one’s role as a nurse emanates from the motivation to care. Nurses have certain expectations of their care-giving role (Ball et al. 2009). One motivation is the social act that Käppeli (2005) describes as a traditional covenant rooted in religion and ethics, known as *caring relationship*. The phenomenon of nurses caring about patients is strongly connected with a reflection of their own motives and involves developing attitudes of generosity, charity, and compassion (de Raeve 2002). The patients’ trust in nurses and their skills warrants a response from the nurses “to care about and not just for the patient” (de Raeve 2002, p. 161). The nurses working in homecare services state exactly this, that they primarily focus on the human first and then on the person being ill (Büscher 2007).

The interest to form a close relationship can be connected with the motivation to care (Duppel 2005). The forming of “close” relationships can contribute to a nurse’s (job) satisfaction (Billeter-Koponen and Fredén 2005; Dowling 2006; Hechinger 2016; Oresland et al. 2008; Ball et al. 2009). The nurses often experience emotional attachment and appreciate human contact either as a reward or as something that adds meaning to their job (Karner 1998; Ball et al. 2009). Most nurses have an ideal in mind of how they would like to form their relationship with patients (Hechinger 2016).

When a nurse cannot improve a patient’s situation, and therefore, fails to meet their personal aspirations—they experience frustration, guilt, and regret (Ball et al. 2009; Bridges et al. 2013). The feelings of failure may also emerge because of a perceived lack of competence in terms of interacting with the patient (Suikkala and Leino-Kilpi 2005). Angel and Vatne (2016) illustrate how the nurse’s perception of a “good nurse” is threatened when he/she fails to provide “proper” care, thus displaying the nurse’s vulnerability. Conversely, nurses tend to experience satisfaction when the care they provide corresponds to their personal aspirations, thus promoting feelings of having done something good (Bridges et al. 2013; Ball et al. 2009; Billeter-Koponen and Fredén 2005). Hence, nurse–patient relationships are a meaningful part that contributes to nurses’ ability to understand their own role.

Due to various demands, nurses experience dissonance in terms of raised expectations (MacIntosh 2003; Hem and Heggen 2003; Oresland et al. 2008; Aranda and Street 1999). A nurse can be described as being constantly torn between professional expectations and human qualities (Aranda and Street 1999; Hem and Heggen 2003; Karner 1998; Oresland et al. 2008; Pohlmann 2005). Hart et al. (2014) refer to challenging work environments and experienced dissonance as contributing factors of resiliency in nurses. An understanding of the aspects that merge into one’s multi-being can support nurses in reconciling the experienced conflicts.

## The patient as a multi-being

**Fig. 2:**
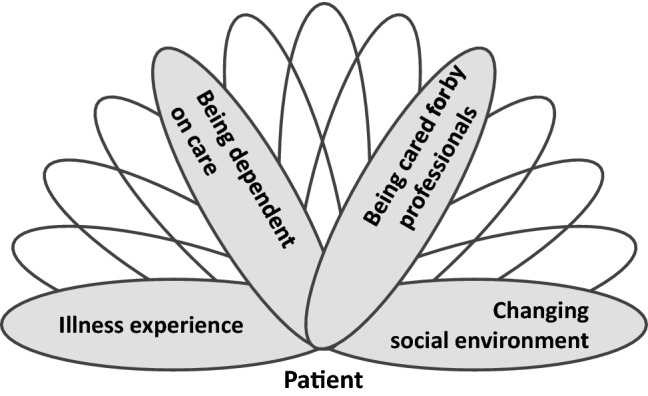
Schematic diagram of patients as multi-being. Own diagram: 2019

The patient’s multi-being is mainly shaped through experiences of illness, the dependence on care, receiving care from professionals, and the changing social environment during the course of homecare (Fig. 2). The following section will focus on these aspects.

### Patient’s experience of illness and impairment

In Gergen’s (2011) terms individuals can *become* “patients” through experiencing this role as children, adults, or elderly persons. Each patient has a unique life story based on a certain background such as the family, the place where he/she was raised, culture, and religion. Each relationship in the patient’s life leaves a residue of potentials. So, at the time of falling ill, the patient is embedded with a multiplicity of different relationships from which he/she has potentials for action at hand.

The main aspect in the patient’s multi-being is the illness or impairment. They tend to experience stimuli differently when suffering from a lack of perception (coma), changed perception (lost hearing, hemiparesis), or distorted perception (hallucinations). Moreover, the reaction to stimuli may vary. The patients may find themselves in exceptional physiological, psychological, and/or sociological conditions owing to an illness that has emerged through a wide range of symptoms and consequences in social life. These conditions have a certain meaning for the individual (Ujhely 1968). The individual’s experience of illness and impairment is shaped by the cultural construct of the society in which he/she lives (Gergen 2015; Charmaz 2000). Gergen (2011, 2015) demonstrates this construction based on the increasing number of mental illnesses since the diagnosis depends on the actual taxonomy. Certain behaviours can be categorized as mental illness as was the case with homosexuality, which is now no longer labelled as a disorder. This illustrates how the perspectives within a society are redirected or altered. Applying Gergen’s (2011) considerations, patients elaborate their own meaning of illness not only from their past experiences but from reactions of others to their illness, that means from the constructed meaning that society attaches onto the illness. This includes any potential reactions the patient may imagine based on his/her knowledge as gained from others or the media in the past.

Mostly, patients in homecare suffer from chronic illnesses. These are often perceived as disrupting the patient’s life (Bury 1982). More than acute illnesses, it is chronic illness that relate to experiences of social, interactional, and existential problems such as identity questions and the reconstruction of one’s self (Charmaz 2000). Patients suffering from chronic illness experience different phases throughout their lives. Fringer et al. (2018) describe that patients as well as their family members experience a wide range of transitions which begins with the onset of symptoms until the patient’s death. A transition is, at first, experienced unconsciously until the occurrence of a crisis. This, as they illustrate, leads the patient and family members to perceive the situation consciously and develop strategies to maintain normality. The patients have to adapt to new life situations. They do so through illness work, everyday life work, and biographical work (Corbin and Strauss 1985). Summing up, the experience of illness is unique to every individual and is based on the patient’s past relationships along with the cultural construct of the actual illness or impairment.

### Being dependent on care

Patients with chronic illnesses may have to be dependent on care for various reasons such as accidents, acute events, or impairments. The experience of care dependency involves relationships with doctors as well as altered relationships with family members and other persons. Such experiences enable new opportunities to absorb potentials and thus, changes the patient’s multi-being. The informal caregivers and the patient’s family play an important role in the patient’s social environment and these relationships form part of his/her multi-being. In this context, Gergen (2011) speaks of the co-creation of shared realities. Over the years, families have negotiated language and action to establish reliable realities, values, and actions that yield trust. However, these previously secure realities are now threatened, which in this case—is because of the dependency on care.

Since the patients cannot execute certain actions any longer, they have to rely on the help of caregivers to carry out these actions. Often, the patients are dependent on caregivers having to anticipate their needs—particularly in cases where these needs cannot be expressed clearly. They also have to rely on others to make decisions for their own welfare (Ujhely 1968). Angel and Vatne (2016) state that the patient’s vulnerability derives from the illness as a threat to their physical, mental, social, and existential behaviours that results from the dependency on care. In their opinion, the patient’s openness can be a source of harm if the care received has a negative effect.

Informal caregivers feel challenged with having to face new life circumstances (Büscher 2007). In such a scenario, the family members such as the husband, wife, and/or children have to take on the role of caregivers. As Gergen (2011) illustrates the parties involved in the relationship have to coordinate themselves to adjust to this new situation. This could refer to changes in the patient’s physicality which may then cause the pattern of communication and mutual understanding (specifically) to change, too. It can be a challenge for informal caregivers to assess the competing demands such as personal beliefs and values, the patient’s needs and preferences as well as the anticipated helpfulness of a homecare service which leads to them having precise ideas and hopes about the nurses’ potential work (Büscher 2007).

### Being cared for by professionals

The decision to use a homecare service is mostly made by the patient and relatives. Such a service provides facilitation directed towards the patients (Holmberg et al. 2012), however the informal caregivers may regard homecare nurses as both a relief and a burden (Büscher 2007).

Individuals who experience care for the first time do not know what to expect and are unfamiliar with nurses, their language, and behaviours (Ashworth et al. 1992). In the initial stage of the nurse–patient relationship, the patients tend to have greater confidence in the nursing agency that sends the nurse rather than trusting the particular nurse assigned to them (de Raeve 2002). Although they may not be able to judge whether the nurse is doing an effective job technically, they may still trust the nurse’s skills (Holmberg et al. 2012; de Raeve 2002). Subsequently, the trust is dependent on the patient’s vulnerability and dependency on the nurse. From having to trust in the beginning trust is built in a dynamic reciprocal process (Dinç and Gastmans 2013).

Primarily, patients want to be seen as individuals and then as sick persons (Holmberg et al. 2012; Büscher 2007). Both, patients and relatives place emphasis on the continuity of having only a few nurses assigned for their care, because discontinuity as an experience causes distress (Holmberg et al. 2012; Büscher 2007; England and Dyck 2011). The patients also appreciate social conversations with their nurses about topics other than their illness. In addition, they value the time taken by nurses to engage in social intercourse (Holmberg et al. 2012; Hechinger 2016). The economic forces of the healthcare system directly affect the experiences of patients and relatives who do not want to become victims of the nurses’ working conditions and time constraints (Büscher 2007; Holmberg et al. 2012).

The impending encounter between a patient and nurse is usually accompanied with certain expectations and fears. While nurses know the context of homecare, patients may only have an idea of professional care, not having used homecare services before. Gergen (2011) illustrates that when two multi-beings meet, they primarily use *scenarios of civility* that comprise familiar patterns of coordination, like greeting each other, saying “please” and “thank you”, or talking about the weather. These are the basic protocols of interaction. Beyond this, however, there are patterns used in a certain context such as the homecare setting. Those *context*-*specific scenarios* comprise rules of relating that are comprehensible but cannot necessarily be transferred onto another context. As the patient is not used to professional care in the homecare context, his/her already-known scenarios do not quite fit. Therefore, the nurse and patient must create their own scenario that is based on their known patterns in order to coordinate in their relationship. “In doing so, we establish a minimally predictable world” (Gergen 2011, p. 152) that breeds trust so that the patient knows what to expect of homecare.

### Changing social environment

The extent of care dependency also causes the patients to experience changes in their social environment. Most often, they are not able to leave their homes without assistance. Nevertheless, as Holmberg et al. (2012) points out, patients try to exert independence and self-determination while being cared for in their own homes. They expect their homes and privacy to be treated with respect. As it is their own home, the patient could deny a nurse entry if he/she behaves in a disrespectful manner. Patients who receive long-term care still consider it as a disruption of their experiences and practices within the home (Angus et al. 2005). Because of the increasing dependency on care and the necessity of homecare services, the patients have to reconfigure their homes leading to inevitable changes, sometimes at the cost of privacy (Angus et al. 2005; England and Dyck 2011). Patients have been “engaged in improvisatory social practices that reflect[ed] their ambiguous and changing habitus or social location” (Angus et al. 2005, p. 169). However, patients do not consider the nurses as guests but as professionals employed to carry out expected tasks in their homes, and therefore, do not act as hosts (Holmberg et al. 2012). Still, they adjust their household routines to accommodate the homecare professionals (England and Dyck 2011; Holmberg et al. 2012). When patients are cared for, their social environment extends to include the nurse and the associated relationship (Schroeter 2008). Especially in long-term care, the nurses become part of the patients’ meaning-in-life as this relationship is perceived as a major aspect of care (Haugan 2014). Moreover, the patients consider their nurses as if they were part of their family and friends (Karner 1998; Hechinger 2016; Mok and Chiu 2004).

Summed up, it can be stated that patients in homecare are in a continuous process of adaptation to their current situation since they experience illness, dependency on care, and a changing social environment. Applying Gergen’s (2011) considerations, each patient has a unique life-story with a multiplicity of different relationships that equip him/her with countless potentials. Getting ill and experiencing dependency on care enables new or changing relationships, and new potentials. The patients find it challenging to be dependent on care, especially having to rely on professional care in their own homes. They have to coordinate with the nurses to build a harmonious relationship while at the same time, coping with triggered emotions associated with having to accept care from others and therefore, being a burden (Delmar 2012). It is important for them to maintain normalcy and their autonomy (Fringer et al. 2018; Holmberg et al. 2012). Although the patients maybe in a vulnerable position due to dependency, they still have certain expectations of nurses. Coping with care dependency becomes easier when the patients are able to rely on nurses and form a friendly and pleasant relationship with them.

## Relationship between nurses and patients as multi-beings in home care

**Fig. 3:**
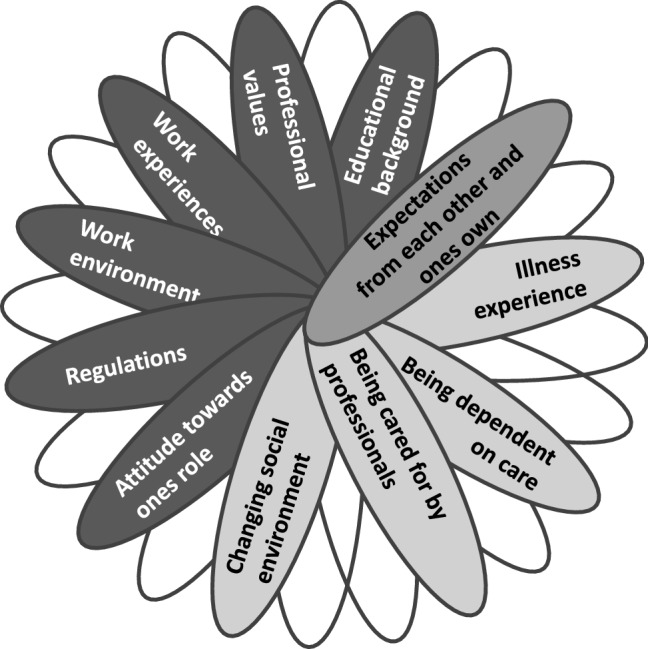
Encounter between the nurse’s and the patient’s multi-being. Own diagram: 2019

The multi-being of nurses and patients has been explained to be constituted by countless potentials that have resulted from countless past relationships. When the two parties meet, they engage only some parts of their multi-being while the other parts remain hidden (Fig. 3). Though the discussed aspects are similar from one nurse or patient to another, the gained potentials differ. This illustrates the heterogeneity and uniqueness of each nurse–patient relationship.

From a social-constructionist perspective the word “relationship” has been given a meaning through a process of co-action (Gergen 2011, 2015). This point of view refers to Wittgenstein’s (2009) understanding that meaning derives from the very use within human interchange. The connotation that an individual has in mind when speaking of “a relationship” depends on the context in which the word is used. The meaning of a word can change since it is construed from the reactions of the individuals to whom it is expressed. Regarding the *nurse*–*patient relationship,* its meaning is a social construction which can change through the years just as different understandings occur in different cultural contexts. Applying Gergen’s (2011) considerations, the nurse’s multi-being is deeply embedded based on the specific development of the nursing profession in a country and in its specific cultural context, thereby. To some extent, German- and English-speaking countries differ in how the nursing profession is organized in regard to professional boards or associations, nursing formations, regulations, and underlying laws. As there are no mandatory guidelines in German-speaking countries so far, the nurses rely on other resources such as the regulations established by their employers; their experiences and learnings, but mainly their personal accounts of how nurse–patient relationships are formed (Hechinger 2016; Büscher 2007). So, it is not beneficial to judge such a nurse’s behaviour from a lens that provides a nursing context of over- or under-involved behaviour as applicable in English-speaking countries. Discussions within the nursing profession of German-speaking countries should promote both, professionalism of the individual and of nursing as a profession.

The nurse and patient meet each other in the context of a homecare service drawn through a contract, which is precise in defining the provided services. While patients experience one changing social environment; nurses, on the other hand, have to negotiate with several individual realities as they experience several different caring contexts relating to different individuals (Büscher 2007). As Blumer (1986) states meaning is constructed in and through interaction. The nurse–patient relationship is a continuous interaction that shapes reality as perceived by each participant. Each action in this relationship gains its meaning from the response action of the other. However, this does not necessarily imply that the meaning given to the words is similar to what the person actually wanted to express, and this could provoke misunderstandings in the conversation. Furthermore, one party could attribute a higher personal value to the nurse–patient relationship than the other by constructing a divergent meaning. Gergen (2011) outlines that a relationship in itself has no value as initially it has to be co-created in terms of confluence with the involved persons. In this context, he also speaks of bonding relationships. To be “bonded” is to be closely connected on an emotional level in a mutually defined manner. He discusses an example of employees who are expected to work using reason, but at the same time, businesses require employees to be dedicated and care about their work. If this is transferred onto the nursing context, nurses are expected to “care for” the patient which refers to attending to his/her bodily needs, however they often voluntarily “care about” their patients (England and Dyck 2011; de Raeve 2002). The expressions of emotional attachment and gratitude can provide nurses with a rewarding experience of appreciation (Karner 1998; Ball et al. 2009). As a result, the nurse–patient relationship can be perceived as one where a bond is formed.

When persons relate to each other and have bonded in a relationship, they stifle impulses and suppress other potentials so as to keep the relationship going (Gergen 2011). Through the course of their interactions, these nurses and patients choose to reveal and/or conceal personal aspects that could contribute to the relationship (Aranda and Street 1999). They create a “we” that differentiates them from “the others”. But, therein lies a threat, as Gergen (2011) illustrates that the bonded relationship is only a new form of bounded entity. It is up to the participants of the very relationships to enter into a mutual dialogue in order to relate and avoid separation. Nevertheless, since the participants are evolving continuously it becomes challenging to maintain the relationship. Furthermore, the nurses and patients are each involved in various relationships simultaneously, which gives rise to new potentials to be absorbed thus changing the multi-being. Therefore, by allowing the newly absorbed potentials to be applied in the nurse–patient relationship. Angel and Vatne (2016) state that the demanding nature of caring relationships should be acknowledged as “the core in their vulnerability lies in the possibility to be the persons they both want to be, and the persons they have not yet become” (p. 1435).

According to Gergen (2011) cause and effect are reciprocally defined. Thus, the nurses, through an action, cannot have a self-contained effect on patients without being affected, and vice versa. The nurse–patient relationship is described as an intersubjective development that is mutually constructed (Tarlier 2004; Aranda and Street 1999). When nurses try to actively form a relationship, their words and actions are always dependent on the reaction of the patient. Based on the patient’s reaction such as his/her words, gestures, and actions—meaning is created. There are various possibilities of how a patient could react to something the nurse proposes, such as amusement, understanding, indifference, taking offence, or anger. Consequently, the nurse–patient relationship can only be formed through the joint effort and never alone. Karner (1998) describes that “negotiating strangerness into familial fictive kin is a social process played out by both actors” (p. 75) and illustrates a dyadic process. This consideration is reinforced by the nurses and patients who describe their relationship as if they were part of the family and friends (Ball et al. 2009; Mok and Chiu 2004; Karner 1998). Therefore, Crowe (2000) proposes an altered view on the nurse–patient relationship that acknowledges the active participation of both parties. Gergen (2011) concludes that “we may abandon the view that those around us cause our actions. Others are not the causes nor we their effects. Rather, in whatever we think, remember, create, and feel, we *participate* in relationship” (p. 397).

The process of nurse–patient relationship is formed by the expectations of both parties (Wiechula et al. 2016). The patients have traditionally-derived role expectations of what the nurse should fulfil. They wish to receive care that display the attitudes of involvement, commitment, and concern instead of just routine nursing care that is provided in an impersonal manner (Attree 2001). They expect nurses to be compassionate, indulgent, and caring while at the same time displaying professional characteristics such as competency, honesty, sincerity, and trustworthiness (Wiechula et al. 2016; Ozaras and Abaan 2018). In addition to patients wanting to be respected as individuals (Ozaras and Abaan 2018; Holmberg et al. 2012) they also expect to be understood and listened to without any judgements (Cleary et al. 1999).

The nurses also desire to be respected as persons with their specific character traits (Büscher 2007; Hechinger 2016). The nurse–patient relationship “is perceived to be dependent on the nurse’s ability to be ‘present’ in the relationship, that is to bring aspects of themselves to the relationship (rather than adopting a work persona)” (Bridges et al. 2013, p. 764). The nurses also express the need to get to know the patient as an individual (Wiechula et al. 2016). Tarlier (2004) uses the term “responsive relationships” in order to describe the nurse–patient relationships which encompass respect, trust, and mutuality. Tarlier (2004) explicates that these relationships are based on the nurses’ personal and public moral knowledge, and thus their ethical nursing knowledge.

The above-mentioned reflections focus on the special aspects referring to the individuals’ multi-beings in terms of their roles as nurses and patients. Therefore, apart from professional aspects, it seems necessary to consider the personal aspects of the nurse’s multi-being, too. The core of the nursing profession is to provide care in a sensitive and empathetic manner; it is not a profession that can be replaced by being cared for automatically. For Green (2013) the culture of bodywork in terms of touching can also “touch” nurses and patients, and it “distinguishes us from inanimate objects” (p. 251). The word has a double meaning in this context. She also sees touching as a constitutive element of our personhood and our relationships with other persons. Consequently, the attitude of caring as a nurse cannot be separated from the individual’s identity as a human being. Touch can convey the nurses’ feelings such as comfort. As personal characteristics are an important part of the nurses’ multi-being, they bring these characteristics into their work (Bridges et al. 2013; Hechinger 2016; Büscher 2007). This acts as the central consideration, since both nurses and patients are, first and foremost, human beings and the potentials of their multi-beings play an important role in the context of their respective roles as nurses and patients. So, to sum up the considerations so far, it is possible to state discrepancies in the nurse-patient relationship, originating from the multi-beings’ past experiences, in regard to inherent human qualities and the demands of forming a relationship, such as guidelines or codes of conduct, *and* the actual relationship based on the framework conditions.

## Conclusions

So far, the complexity of the nurses’ and patients’ multi-beings have been outlined. Specific aspects pertaining to the provision of services in the patient’s home have been considered as well. The multi-beings of each nurse and patient are constituted by potentials from various past relationships. When two multi-beings, from a divergent context, eventually meet in an encounter they both have to make efforts to promote a mutual relationship. The concept of nurses and patients as multi-beings has enabled a differentiated perspective in terms of their individual constitution, which has contributed towards the nurse–patient relationship in homecare. The way in which nurses and patients gain potentials has been clarified; the importance of the role played by the individual’s constitution within the complexity of the nurse–patient relationship has also been discussed. Furthermore, an understanding of the nurse’s role and the limited scope of his/her action within the relationship could be gained. Gergen (2011) negates the concept of free will which means that decision making is not an independent process. Instead, the decisions are an outcome of the nurses having absorbed countless potentials in the past that are now incorporated in their individual selves, and which are at hand in a current interaction as potential actions. Thus, this implies that nurses possess a limited scope of action to make their decisions. Even though the potentials may have been absorbed well before taking on the role of nurse or patient, these cannot be hidden because they are part of the nurse’s and patient’s multi-beings. Such potentials, are among others, friendly attention, the ability to listen, to touch or comfort. When transferring these notions of multi-being onto the German homecare setting, the nurse’s self-responsibility to form an independent relationship with the patient is more comprehensible; as in the absence of mandatory guidelines, the nurses mainly refer to the remaining aspects of their multi-being (see Fig. 3). Thus, their multi-being comprising their potentials as humans as well as their potentials as nurses—such as educational background, professional values, work experiences, work environment, and their attitude towards their own role—become more important.

The described notions illustrate the complexity of the nurse–patient relationship wherein the nurses and patients as individual complex multi-beings act and form a relationship, together. Understanding this concept can help nurses to comprehend different behaviours, attitudes, or opinions as potentials of their own selves. It can encourage them to explore the contributing aspects of their own or patient’s multi-being that may contribute to the joint relationship. Examining the possible aspects can lead to the reflection and awareness of the relational character of the multi-being and subsequently, the individual character of the nurse–patient relationship. As a consequence, nurses can attempt to form conscious relationships with their patients.

Gergen’s (2011) considerations promote a comprehensive understanding of the individual’s constitution that executes the role of nurse or patient along with the circumstances under which the nurse-patient relationship is formed. This article can be considered as a preliminary attempt to explore the interrelationship of nurses and patients as multi-beings. Future studies might, for example deal with the topic of “difficult” nurse–patient relationships. Research should concentrate on the actual encounter between nurses and patients using the concept of multi-being. The constitution of the relationship should be explored along with studying how the nurse–patient relationship is formed, especially in the homecare setting. As such a perspective is currently lacking this will be focused on in a planned research project.
